# Editorial: Recent advances on grapevine-microbe interactions: From signal perception to resistance response, volume II

**DOI:** 10.3389/fpls.2022.1069886

**Published:** 2022-11-08

**Authors:** Michele Perazzolli, Aziz Aziz, David Gramaje, Ana Margarida Fortes, Dario Cantu

**Affiliations:** ^1^ Center Agriculture Food Environment (C3A), University of Trento, San Michele all’Adige, Italy; ^2^ Research and Innovation Centre, Fondazione Edmund Mach, San Michele all’Adige, Italy; ^3^ University of Reims Champagne-Ardenne, Induced Resistance and Plant Bioprotection, USC INRAE 1488, Reims Cedex, France; ^4^ Instituto de Ciencias de la Vid y del Vino (ICVV), Consejo Superior de Investigaciones Científicas Universidad de la Rioja, Logroño, Spain; ^5^ BioISI – Instituto de Biosistemas e Ciências Integrativas, Faculdade de Ciências, Universidade de Lisboa, Lisboa, Portugal; ^6^ Department of Viticulture and Enology, University of California Davis, Davis, CA, United States

**Keywords:** grapevine, *Vitis vinifera* L, disease control, induced resistance, breeding, biocontrol, plant-microbiome interactions

Grapevine (*Vitis vinifera* L.) is one of the most economically important perennial crops and is susceptible to a wide array of diseases caused by pathogenic oomycetes, fungi, bacteria, and viruses that are responsible for negative effects on plant growth, fruit production, and fruit quality. Downy mildew caused by *Plasmopara viticola*, powdery mildew caused by *Erysiphe necator*, and trunk diseases involving multiple fungi, remain the most threatening in grapevine-producing countries. Frequent applications of fungicides are used to control grapevine pathogens, with consequent risks of environmental pollution and toxicity. In recent years, there has been an increasing interest in developing more sustainable control strategies, such as selecting resistant grapevine genotypes and developing biofungicides based on biocontrol agents and resistance inducers. This second volume of Research Topic covers the most recent knowledge in the molecular mechanisms of grapevine-microbe interactions and provides an overview of (i) resistance traits for breeding of new resistant cultivars, (ii) molecular and metabolic basis of grapevine resistance to pathogens; (iii) innovative products and strategies for disease management; and (iv) possible risks of pathogen outbreaks.

Breeding for resistant genotypes represents a promising alternative to reduce grapevine diseases. Some resistance loci/genes are characterized and others are currently being studied to gain a better understanding of the mechanisms of resistance and develop cultivars with increased tolerance against the major pathogens, such as powdery mildew and downy mildew. The resistance (R) loci *Run1* and *Ren1* confer resistance to powdery mildew. Sosa-Zuniga et al. studied the physiology of grapevines carrying *Run1/Ren1* in the presence of *E. necator* infection and reported that efficient physiological and biochemical parameters are maintained during the interaction. The authors concluded that the defense response triggered by *Run1/Ren1* prevents the development of powdery mildew infection with no energy costs for the plant. Stacking resistance genes is a key strategy in grapevine breeding. Ruiz-García et al. identified three genotypes with strong combined resistance to powdery and downy mildew in a population of 28 genotypes obtained from crosses between grapevine cultivar (cv.) Monastrell and cv. Regent. Using functional and molecular assays, the authors confirmed the presence of resistance-associated alleles of simple sequence repeat (SSR) markers for the loci *Rpv3* and *Ren3* responsible for resistance to downy and powdery mildew, respectively. Štambuk et al. showed that the constitutive profile of polyphenolic compounds in grapevine leaves contributes to the discrimination of different resistance levels to downy mildew in Croatian grapevine germplasm. In particular, the most abundant compounds detected in resistant Croatian genotypes were myricetin-3-O-glucoside, quercetin-3-Ogalactoside, quercetin-3-O-glucoside, kaempferol-3-O-glucoside, caftaric acid, gallocatechin, procyanidin B1, and piceatannol before inoculation and 24, 48, and 96 h post-inoculation. The high accumulation of piceatannol and total stilbenes was also associated with the response of a completely resistant genotype (*V. riparia*) to *P. viticola*.

The identification of novel products, the development of efficient fungicide formulations, and the appropriate prediction/modeling of pathogen infection are also important for sustainable control strategies against grapevine diseases. Rashad et al. showed that spray application of silica nanoparticles (SiNPs) reduces downy mildew severity together with the upregulation of the genes coding for jasmonic acid and ethylene responsive factor 3, pathogenesis-related-protein 1, β-1,3-glucanase, peroxidase, and chitinase. SiNPs treatments also improved shoot length, fruit yield, berry quality, the content of phenolic and ascorbic acid, and the activity of peroxidase and polyphenol oxidase enzymes. However, cytotoxic and genotoxic effects of SiNPs were pointed out, suggesting that the assessment of optimal dosage of application, toxicity, and risk for the environment are required to further develop this method for downy mildew control. In addition to new active molecules, novel formulations can also improve fungicide efficacy and spectrum of activity. Battiston et al. showed that copper-based treatments, formulated with hydroxyapatite, inhibit the esca-associated fungus *Phaeoacremonium minimum* in grapevine propagation material. The authors reported that hydroxyapatite reduces the fungicide effect of copper, but it can display fungistatic properties against *P. minimum* and increase copper persistence in the grapevine-treated tissues. They also suggested that hydroxyapatite could modify the balance between the fungicidal/fungistatic effects and the plant defense elicitation of copper. Gonzalez-Dominguez et al. developed and validated an epidemiological model that predicts *Diaporthe ampelina* (synonym *Phomopsis viticola*) infections responsible for Phomopsis cane and leaf spot of grapevine (also known as ‘excoriose’). Further integration of the model into a decision supporting system will help growers to determine when a fungicide application is needed, according to the epidemiological models, and to optimize fungicide applications. However, the reduction of chemical treatments and the intensification of crop management practices can potentially lead to an increase in the outbreaks of other pathogens, such as the causal agents of grapevine trunk diseases. A review article by Azevedo-Nogueira et al. explored the state-of-the-art procedures for the detection and identification of grapevine trunk diseases, highlighting that the development of new technologies is required to precisely detect the presence of fungal pathogens and readily apply phytosanitary measures and/or proceed to plant removal. The authors suggest that new methods for the precise identification and quantification of grapevine trunk pathogens are particularly important to allow efficient testing of propagation material in nurseries and to avoid the spread of fungal inoculum throughout wine regions.

The papers included in this Research Topic highlighted the growing effort in the investigation of disease control methods against grapevine pathogens. Approaches include breeding programs for new resistant cultivars, understanding of resistance mechanisms, testing of innovative products and formulations for disease management, and evaluation of epidemiological models and risks of emerging pathogens. Further studies in these fundamental and applied Research Topics will allow a better understanding of molecular regulations of grapevine-pathogen interactions and the identification of practical solutions for growers. However, the solution will come possibly from using multidisciplinary approaches and integrated multi-omic analyses.

## Author contributions

All authors listed have made a substantial, direct, and intellectual contribution to the work and approved it for publication.

## Conflict of interest

The authors declare that the research was conducted in the absence of any commercial or financial relationships that could be construed as a potential conflict of interest.

## Publisher’s note

All claims expressed in this article are solely those of the authors and do not necessarily represent those of their affiliated organizations, or those of the publisher, the editors and the reviewers. Any product that may be evaluated in this article, or claim that may be made by its manufacturer, is not guaranteed or endorsed by the publisher.

